# Reconstructing the Life of an Unknown (ca. 500 Years-Old South American Inca) Mummy – Multidisciplinary Study of a Peruvian Inca Mummy Suggests Severe Chagas Disease and Ritual Homicide

**DOI:** 10.1371/journal.pone.0089528

**Published:** 2014-02-26

**Authors:** Stephanie Panzer, Oliver Peschel, Brigitte Haas-Gebhard, Beatrice E. Bachmeier, Carsten M. Pusch, Andreas G. Nerlich

**Affiliations:** 1 Department of Radiology, Trauma Center Murnau, Murnau, Germany and Biomechanics Laboratory, Paracelsus Medical University Salzburg and Trauma Center Murnau, Murnau, Germany; 2 Institute of Legal Medicine, Ludwig-Maximilians University, Munich, Germany; 3 Bavarian State Archaeological Collection and Museum, Munich, Germany; 4 Institute of Laboratory Medicine, Ludwig-Maximilians University Munich, Germany; 5 Institute of Anthropology and Human Genetics, Eberhard-Karls-University Tübingen, Germany; 6 Institute of Pathology, Academic Clinic München-Bogenhausen, Munich, Germany; University of Florence, Italy

## Abstract

The paleopathological, paleoradiological, histological, molecular and forensic investigation of a female mummy (radiocarbon dated 1451–1642 AD) provides circumstantial evidence for massive skull trauma affecting a young adult female individual shortly before death along with chronic infection by *Trypanosoma cruzi* (Chagas disease). The mummy (initially assumed to be a German bog body) was localized by stable isotope analysis to South America at/near the Peruvian/Northern Chilean coast line. This is further supported by New World camelid fibers attached to her plaits, typical Inca-type skull deformation and the type of Wormian bone at her occiput. Despite an only small transverse wound of the supraorbital region computed tomography scans show an almost complete destruction of face and frontal skull bones with terrace-like margins, but without evidence for tissue reaction. The type of destruction indicates massive blunt force applied to the center of the face. Stable isotope analysis indicates South American origin: Nitrogen and hydrogen isotope patterns indicate an extraordinarily high marine diet along with C4-plant alimentation which fits best to the coastal area of Pacific South America. A hair strand over the last ten months of her life indicates a shift to a more “terrestric” nutrition pattern suggesting either a move from the coast or a change in her nutrition. Paleoradiology further shows extensive hypertrophy of the heart muscle and a distended large bowel/rectum. Histologically, in the rectum wall massive fibrosis alternates with residual smooth muscle. The latter contains multiple inclusions of small intracellular parasites as confirmed by immunohistochemical and molecular ancient DNA analysis to represent a chronic *Trypanosoma cruzi* infection. This case shows a unique paleopathological setting with massive blunt force trauma to the skull nurturing the hypothesis of a ritual homicide as previously described in South American mummies in an individual that suffered from severe chronic Chagas disease.

## Introduction

The human remains (mummies and skeletons) from previous cultures represent an enormous bioarchive suitable for the reconstruction of living and disease conditions in past populations, including evidence for infectious diseases and violent trauma. The application of modern analytical techniques provides an increasing spectrum of information to be used. Accordingly, the recent significant advances of modern radiological techniques and molecular analysis of various biomolecules (ancient DNA, “proteomics”) and instable as well as stable isotopes (absolute dating, diet, localization of origin) provide an increasing body of information. To this regard, complete mummies are much more informative than mummy parts or only bones [1]. Fine examples for the potential of such studies have exemplarily been recently shown by Hawass et al. [2], [3] in the case of the mummy of Pharaoh Tutanchamun and other royal mummies from ancient Egypt. Accordingly, any such study seems to be helpful for our understanding of the past.

In this report, we describe the interdisciplinary study of a previously unknown mummy brought in the 1900s to Bavaria, Germany, which is now housed in the Bavarian State Archeological Collection. Since no records were available on the origin, life and living conditions, we used a broad panel of techniques to unravel the “life story” of the female individual resulting in an intriguing observation with an unexpected paleopathological and forensic outcome.

## Materials and Methods

The mummy is currently housed in the Bavarian State Archaeological Collection. It is part of the museum since 1970 when it was transferred from the Anatomical Institute of the Ludwig-Maximilians University. There is has been recorded first in 1904. The mummy has no specimen or particular identification number (there exists only the identification number of the Anatomical Institute of the Munich University, #817/1904, and there has been given no new number when transferred to the Bavarian State Archaeological Museum in 1970; since this is, however, the only mummy in that museum it can unambiguously be identified and accessed by other researchers). All studies were undertaken with the full consent of the museum and supervised by one of the co-authors (BH).

**Figure 1:**
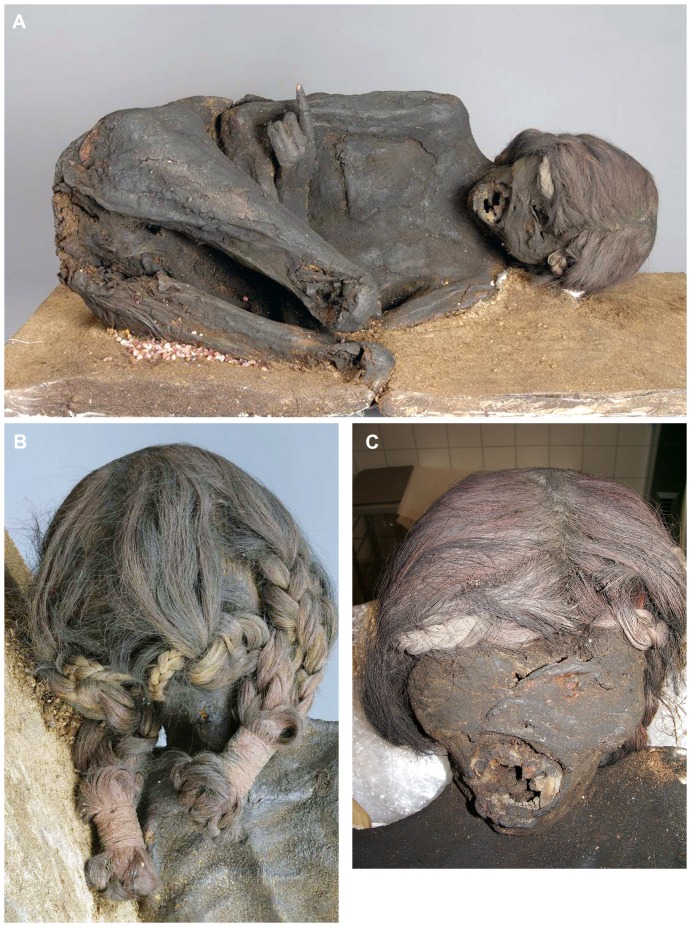
Macroscopic aspect of the mummy. (**A**) Frontal view of the mummy which reveals typical squatting position (although the legs are broken off below both knees). (**B**) External appearance of the hair plaits which are fixed at their ends by tiny ropes of foreign material. (**C**) Detailed view of the mummy’s face. Note the transverse defect above the left eye. Both eyes are closed and covered by skin. The mouth is ovally opened, the frontal teeth are missing.

Whilst the mummy was recorded first in the Anatomic Institute, there is no written evidence available neither about the mummy’s origin nor its way to the Munich University. During World War II, the mummy suffered some damage (especially the loss of both lower legs) during bombing [4]. Due to the dark brown external appearance (Figure 1A), she was tentatively assumed to represent a “bog body” from a moor region in the close surroundings of Munich (e.g. from the so-called “Dachauer Moos”) [4]. Unusually for this assumption, however, were the good preservation of the outer shape and – even more remarkably – the well-preserved structure of bone tissue even in previous radiograms [4]. Furthermore, despite the missing lower legs (that seem to have been torn off more recently), the female is presented in a squat-position and wears long aesthetic plaits which again is highly unusual for individuals of past European populations (Figure 1B). In the 1970ies the mummy was donated to the Bavarian Archeological State Collection, Munich. Since its donation, the female body had been on exhibition for several decades in the collection.

Due to the inconsistencies with a presumed Middle European origin, the collection decided to investigate the mummy in more detail. This work-up consisted of a concise macromorphological and anthropological investigation, a complete body computed tomography (CT) scan, analysis of stable and unstable isotopes, histological analysis of various tissue samples, molecular identification of parasitic ancient DNA (aDNA) and forensic reconstruction of injury.

### Paleopathological and Anthropological Investigation

In a first step, the mummy was subjected to a detailed anthropological and macroscopical inspection. As indicated before, a full body length could not directly be measured due to a lack of both lower legs. Form and shape of the hair/plaits were recorded. The estimation of body height was done by estimating body height from the (radiologically determined) maximum length of the femora and humeri according to the established data sets by Sjovold [5].

### Paleoradiological Analysis

The mummy was thoroughly investigated by full-body CT scan (64-row detector CT, LightSpeed VCT, General Electrics, Milwaukee, Wisconsin, USA) in supine position with slice thickness of 0.625 mm, interval of 0.625 mm, 120 kV and 200 mA in standard algorithm as previously done [6]. Additional three-dimensional and multi-planar reconstructions as well as maximum intensity projections were prepared on the attached workstation (ADW 4.4, General Electrics, Milwaukee, Wisconsin, USA).

### Microscopical Study and Morphological Hair Analysis

For the investigation of the bone and soft tissue structure, first a bone-cartilage sample was taken at the “open end” of the right broken leg. Here the right patella was easily accessible. The sample was subsequently divided into equal halves with one being directly embedded into Epoxy resin and a resin section (4 µm thickness) was cut for Toluidin blue staining. The second half was rehydrated with Ruffer solution, gently decalcified and embedded into paraffin for routine histological staining as previously done [7], [8]. The details of the histomorphological analysis are presented in Text S1.

Furthermore, a full-thickness biopsy was taken from the rectum wall which was divided into one half for histology and one half for subsequent molecular analysis. The histological sample was rehydrated as indicated before. All histological samples were stained with H&E, connective tissue stains, PAS, Grockotts silver staining, Prussian blue and May- Grünwald-Giemsa stain [7]. Furthermore, the histological sample was subjected to an immunohistochemical analysis of particular parasites (*Trypanosoma cruzi*, antibody Acris Antibodies GmbH, Herford Germany). This analysis was performed according to the previously established protocols for immunostainings of rehydrated mummified tissue material [8] (details of tissue preparation and staining are given in Text S1).

An analysis of the mummy’s hair, as well as fibre analysis of the small fibre bundles that fixed the hair at its ends was performed by light microscopy (and in case of the fixing fibers with scanning electron microscopy). The latter was kindly performed by Mrs. Kerstin Gonda, Bayerisches Landeskriminalamt, Munich, Germany.

**Table 1:** Sequence Data of the T. cruzi amplification product.

Qu:	1 CCCCCCTCCCAGGCCACACTGGGGAGGGGTCAGGGTCCGGGACGTCGGCCCCGGGATAGC 60
	||||||||||||||||||||||||||||||||||||||||||||||||||||||||||||
Sb:	220 CCCCCCTCCCAGGCCACACTGGGGAGGGGTCAGGGTCCGGGACGTCGGCCCCGGGATAGC 279
Qu:	61 GCCCTGGACCGCGT 74
	||| ||||| ||||
Sb:	280 GCCGTGGACGGCGT 293

Note: Qu: = Reference sequence; Sb: = Subject tested.

### Unstable and Stable Isotope Analysis

Unstable isotope analysis of ^14^C-radiocarbon was done in order to evaluate the mummy’s actual living period. This was done by the Archeometry Laboratory of the University of Erlangen with calibrated dating evaluation.

Stable isotopes were determined in a hair strand (nitrogen, carbon,) according to well established protocols. Details and background are presented in Text S2. In brief, the hair strand of approximately 10 cm length was carefully removed from the mummy’s scalp, the proximal end was marked, the sample carefully cleaned with distilled water and dissected into 2 cm long segments. The standardized procedure of O’Connell and Hedges [9] was strictly followed. Stable isotope analysis and concentration measurements of nitrogen and carbon were performed using isotope ratio mass spectrometry (IsoAnalytical, Crewe, UK).

### Molecular Investigation of Psychoactive Substances

In a further approach, we tested a strand of mummy hair for the presence of various psychoactive substances, particularly those frequently used in South American settings. Therefore, the strand was carefully dissected, cleaned, and analyzed by GC-MS according to routine testing [10]. Resulting peaks were identified by comparison of elution intervals. Accordingly, the presence of tetrahydrocannabinol, cocaine, heroine and many other psychoactive substances was tested. All tests were kindly performed by D. Thieme, IDAS Kreischa/Dresden, Germany.

**Table 2:** Stable isotope analysis of mummy hair.

Distance from skull	delta^13^C	delta^15^N
0–2 cm	−11.01	23.50
2–4 cm	−12.48	25.12
4–6 cm	−12.54	25.24
6–8 cm	−12.57	24.95
8–10 cm	−12.37	25.11

### Molecular Investigation of Ancient Parasitic DNA

In order to confirm potential infection by *Trypanosoma cruzi* as suggested by paleoradiology and histopathology/immunohistochemistry we subsequently performed a molecular investigation of the second half of the rectum wall sample. Accordingly, the surface of the material was carefully removed under a dissection microscope and the sample was minced and powdered. The tissue powder was then subjected to DNA extraction with subsequent PCR amplification according to established protocols [11], [12]. As an initial test for the presence of amplifyable DNA we targeted the ubiquitous human beta-actin gene (202 bp) as previously performed [12]. Subsequently, further aliquots of the extracted DNA were used for amplification using specific primers for *Trypanosoma cruzi* and subsequent Sanger sequencing. Primers and protocols for thermal cycling have been successfully applied to aDNA research by previous researchers, such as Aufderheide et al. [13]– [15]. Detailed information on the molecular analysis is given in Text S3.

## Results

### Macroscopy and Anthropology

In general, the mummy is excellently preserved and presents macroscopically lying on her left side in a squatting position with both legs broken off below the knees. She is strongly flexed in the hips and both arms are crossed over the abdomen (Figure 1A). The mummy has unambiguous external female genitalia and additionally, the residues of both mammae are clearly visible. Below the crossed arms a transverse defect of approximately 15×0.5 cm length runs across the abdominal wall. The remaining surface is smooth without any defects.

The skull is covered by dense brown to slightly reddish hair that are formed into two long plaits and which are fixed at their ends with tiny ropes (Figure 1B). The mouth is oval opened; the frontal teeth are missing (Figure 1C). Since the dental alveoli are regularly open the loss of teeth is regarded to be post mortal. At the skull front an approximately 7 cm long, slightly oblique defect runs through the soft tissue and opens into the skull cavity (Figure 1C). Both eyes are covered by the eye lids. Otherwise, the skull is externally without further defect.

Since the complete body length could not be determined due to the missing lower legs, we estimated the whole body length anthropologically suggesting 167 cm.

### Paleoradiological Investigation

For further detailed analysis, a whole body CT-scan was performed. This confirmed the outer details described before, but additionally showed significant inner findings.

#### Skeleton

**Figure 2:**
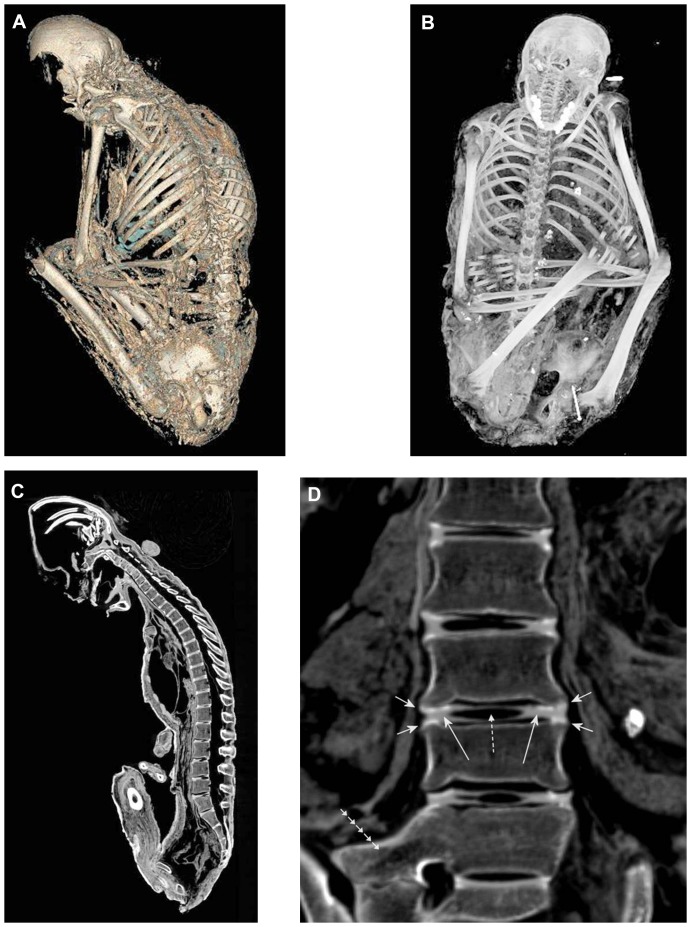
Paleoradiology – The skeleton of the mummy. (**A**) Three dimensional reconstruction and (**B**) maximum intensity projection of the complete mummy giving an overview on the skeleton. (**C**) Sagittal CT reformation image showing the untouched spine and the preservation of thoracic and lumbar intervertebral discs. (**D**) Coronar CT reformation image of the lumbar spine demonstrating incomplete fusion of the apophyses of the lumbar vertebrae (short arrows). The preserved intervertebral discs reveal the outer annulus fibrosus (long arrows) as well as the inner nucleus pulposus that is now replaced by air (dotted arrow). Exemplary inscription of the segment between the third and fourth lumbar vertebra. Constitutional segmentation defect with fusion of the fifth lumbar vertebra and the sacrum on the right side (arrow line).

In general, the skeleton was excellently preserved and the bone structure did not show any conspicuous demineralization (Figure 2). The thoracic skeleton revealed fractures of the eighth, ninth, eleventh rib on the right side, the tenth rib on the left side, and the right acromion and scapula, altogether without any osseous reaction (bone resorption or callus formation). Some joints of the thoracic and pelvic skeleton showed a slight malposition. The vertebral column revealed incomplete fusion of the apophyses (Figure 2D) which suggests, together with other skeletal findings, an age of the mummy between 20 and 25 years. Additionally, the lumbar spine showed a constitutional segmentation defect with fusion of the fifth lumbar vertebra and the sacrum on the right side (Figure 2D).

#### Head

**Figure 3:**
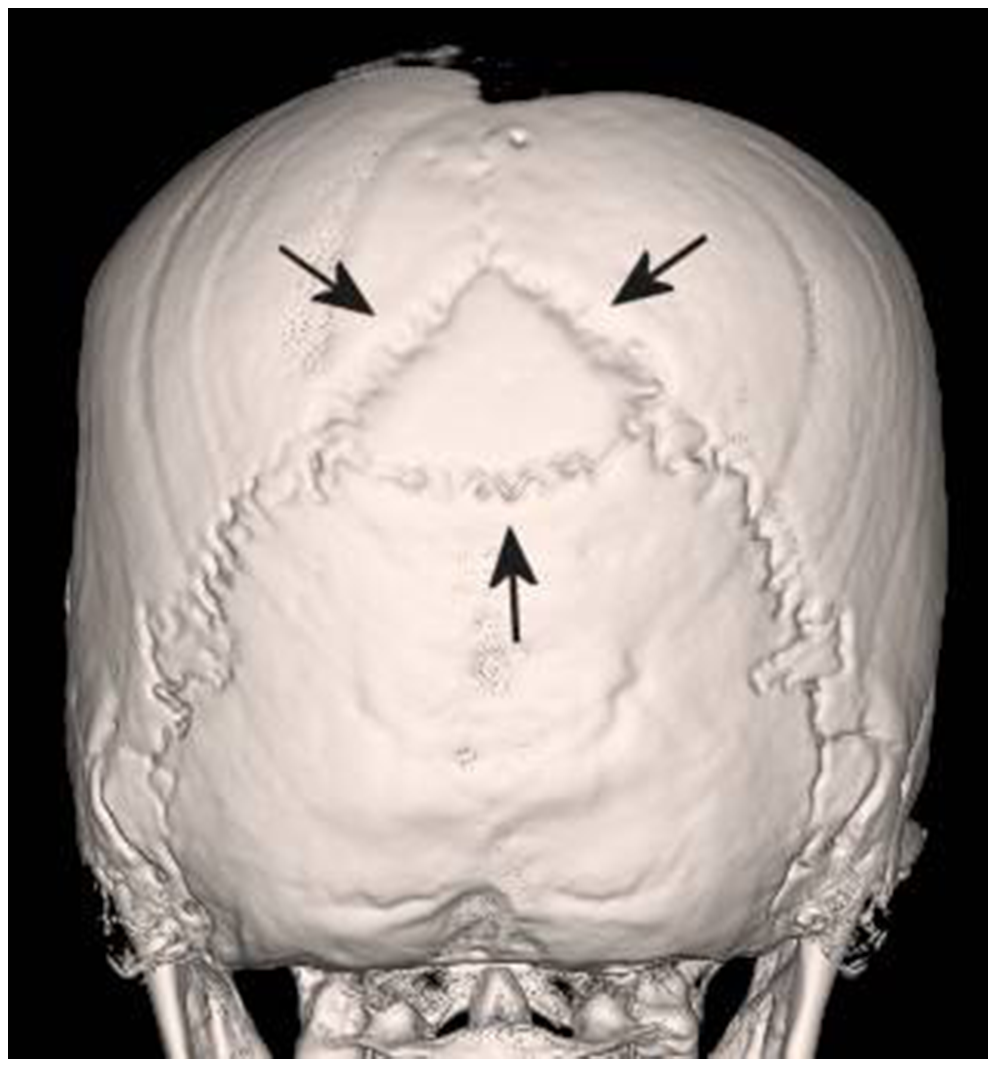
Paleoradiology – The Wormian “Inca” bone. Three-dimensional reconstruction of the head with back view demonstrating an Inca bone. This anatomical variation represents an additional bone in the lambdoid suture. The present type of Inca bone is typically seen in South American populations, but not in European ones.

**Figure 4:**
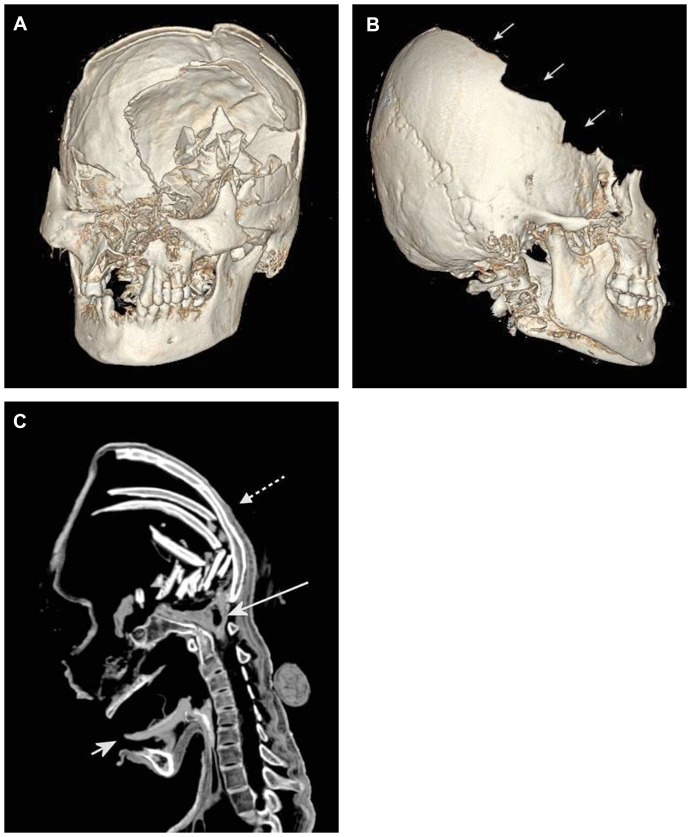
Paleoradiology – Signs of massive craniocerebral injury. (**A, B**) Three-dimensional reconstructions of the head illustrating destruction of the upper and frontal parts of the skull as well as the midface. On the right side “terrace-like”, slightly arching defects of the skull are discernible (arrows in B). (**C**) Sagittal CT reformation image of the head and neck showing numerous bony fragments inside the skull, and in between remnants of brain tissue and possibly of bleeding which accumulated especially in the posterior fossa and foramen magnum (long arrow). Preserved tongue (short arrow) with centrally overlying light seam, presumably representing remnants of bleeding. Note the conspicuous flattening of the preserved occiput (dotted arrow) indicating artificial deformity in the lifetime of the mummy.

The three-dimensional reconstructions of the head revealed an Inca bone (Figure 3) representing an additional bone in the lambdoid suture which can be classified as type III according to the Hanihara and Ishida classification [16]. Furthermore, the preserved occiput showed a conspicuous flattening (Figure 4C) indicating artificial deformity in the lifetime of the mummy [17].

The most remarkable pathological findings were seen in the skull with signs of massive craniocerebral injury (Figure 4). The upper and frontal parts of the skull as well as the midface were completely destroyed and collapsed. On the right side “terrace-like”, slightly arching defects of the remaining skull were discernible (Figure 4B). The maxilla revealed a median cleavage fracture. The mandible presented undamaged. Numerous bony fragments were lying inside the skull, and in between remnants of brain tissue and possibly of bleeding which accumulated especially in the posterior fossa and foramen magnum (Figure 4C). The tongue was preserved and a centrally overlying light seam was recognizable, presumably representing remnants of bleeding (Figure 4C). Several anterior teeth of manible and maxilla were missing. The remaining teeth did not show significant wear. The last tooth on the right side of the manible revealed a small apical granuloma.

Interestingly, a radiopaque metallic foreign body was detected inside the inverted end of the left plait which could be removed for further investigation. It was possibly serving as a pin holder at the site of the small ropes, however, its definite role remained unclear.

#### Internal organs

**Figure 5:**
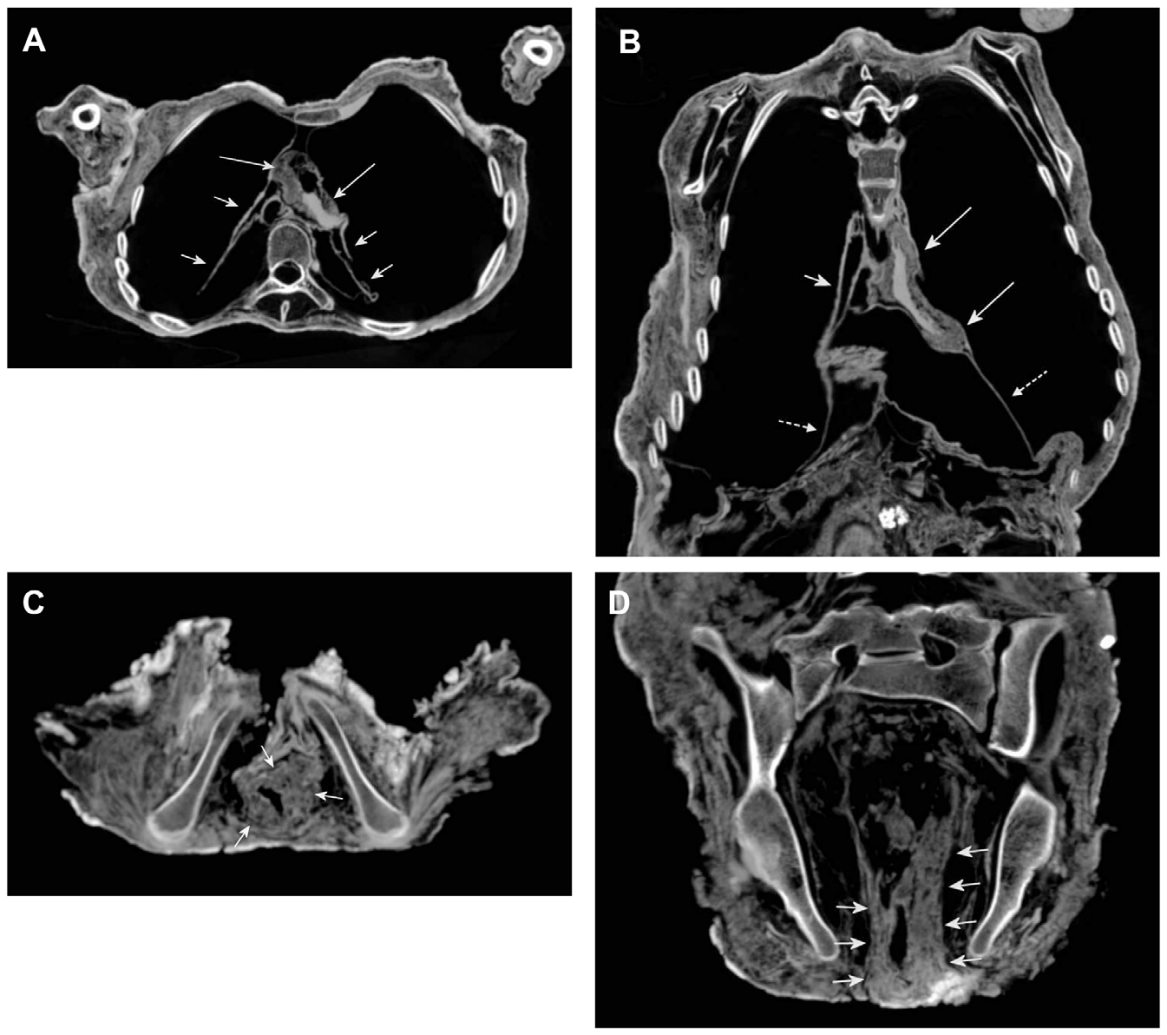
Paleoradiology – Pathologically thickened wall of the heart and rectum. (**A**) Axial and (**B**) coronar CT reformation image of the chest illustrating collapsed lung (short arrows) and a relatively large heart with a markedly thickened wall (long arrows). The heart overlies the diaphragm (dotted arrow in B). The longish hyperdense structure inside the heart represents dried blood. (**C**) Axial and (**D**) coronar CT reformation image of the lesser pelvis demonstrating massive circular thickening of the wall of the rectum (arrows). Centrally, the inflated lumen is discernible. The combination of pathologically thickened wall of the heart and rectum suggested the diagnosis of Chagas disease. For further investigations, minimal destructive biopsy of the rectum was planned on the basis of the CT reformation images.

The excellent preservation status of soft tissues allowed assessment of some internal organs. On both sides of the chest collapsed lung was detectable (Figures 5A, B). Despite postmortal shrinkage, the heart was preserved as relatively large organ, widely overlying the diaphragm with thickening of the muscle wall up to 1.2 cm on the left heart. Inside the heart dried blood was visible as a longish hyperdense structure (Figures 5A, B). The liver was preserved as relatively large organ. The oesophagus was neither thickened nor enlarged, however, the complete abdomen was filled by distended intestinal loops, partially including up to 1.5 cm large calcifications. Furthermore, the intestinal walls especially of the large intestine revealed circular thickening up to 1.2 cm in the region of the rectum. Centrally, the inflated rectal lumen was discernible (Figures 5C, D). The combination of pathologically thickened wall of the heart and rectum suggested the diagnosis of Chagas disease. For further investigations, minimal destructive biopsy of the rectum was planned on the basis of the CT reformation images.

### Radiocarbon Dating (Instable Isotope)

The calibrated radiocarbon dating (^14^C) of the mummy revealed a life time span between AD 1451–1642 (2-sigma range).

### Histomorphology and Hair Analysis

**Figure 6:**
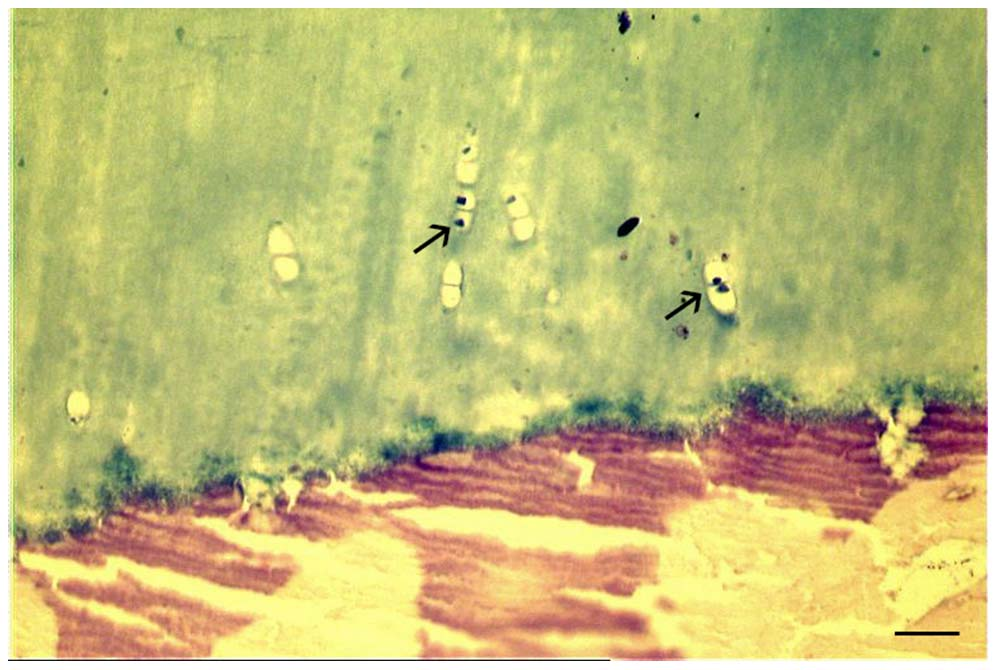
Histology of bone and cartilage. Undecalcified section through the cartilage (upper half) –bone (lower half) transition zone of the patella. Note the excellently well preserved cartilage and bone matrix and residues of nuclear material in some of the chondrocytes (arrows). (Giemsa-May-Grünwald staining, undecalcified section, polyacryl embedding, bar 150 µm).

In order to evaluate the state of conservation and the metabolic status of the individual, we investigated bone and cartilage samples both by undecalcified and decalcified histology including various histochemical stainings (Figure 6). Both approaches showed excellently preserved tissue of bone and cartilage with typical osteocytic or chondrocytic holes, sometimes with clumpy residues of nuclear remains (Figure 6). The cartilage appeared typically structured without clefting or chondrocyte proliferation. Additionally, in undecalcified sections there was no evidence for enhanced metabolic bone turn-over, such as in osteomalacia, hyperparathyroidism or other metabolic conditions affecting bone.

**Figure 7:**
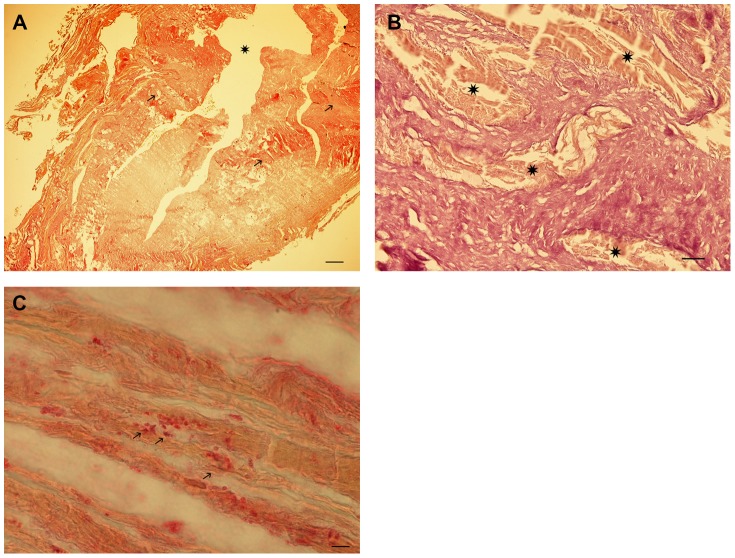
Histology of the rectal wall sample. (**A**) Overview of the specimen showing the lumen of the rectum (asterisk) which is surrounded by the rectal wall with a typical ring-like smooth muscle (arrows). (Haematoxilin and eosin, bar 500 µm). (**B**) A detailed view of the rectal wall (connective tissue stain) shows smooth musculature (asterisks) which is interspersed by broad bundles of collagen providing the clear diagnosis of massive fibrosis. (Van Gieson connective tissue stain, bar 100 µm). (**C**) Immunohistochemical staining with a monospecific antibody against *Trypanosoma cruzi* showing a positive immunostaining of small corpuscular inclusions in smooth muscle cells (arrows). (anti-*Trypanosoma cruzi*, APAAP-staining, bar 10 µm).

Despite the complete lack of the rectal mucosa, the rectal tissue specimen is clearly identified containing a broad zone of fibrosis with intermingled muscle structures (Figure 7). Accordingly, the tissue does no longer contain cell nuclei (Figures 7A and B); however, in the muscle fiber area small round inclusion bodies are seen in high power magnification that are clearly restricted to the muscle residues. The application of special stainings revealed negative results for fungi, bleeding residues and foreign material. In the immunostainings, however, these corpuscular structures are selectively and strongly stained with a monoclonal antibody against *Trypanosoma cruzi* (Figure 7C). The staining pattern is restricted to the light microscopically identified corpuscles. All negative staining controls as well as the fibrosis areas remain unstained.

The hair sample revealed brown pigment residues along the shaft suggesting dark hair colour intra vitam. Furthermore, the mummy’s hair showed typical structure of human hair.

In contrast, the analysis of the small fiber bundles at the ends of the plaits revealed non-human type of hair structure typical to that of camelids; furthermore, the wavy pattern of the hair margins is suggestive of New World camelids (lama, alpaca) (not shown).

### Molecular Ancient DNA Analysis

**Figure 8:**
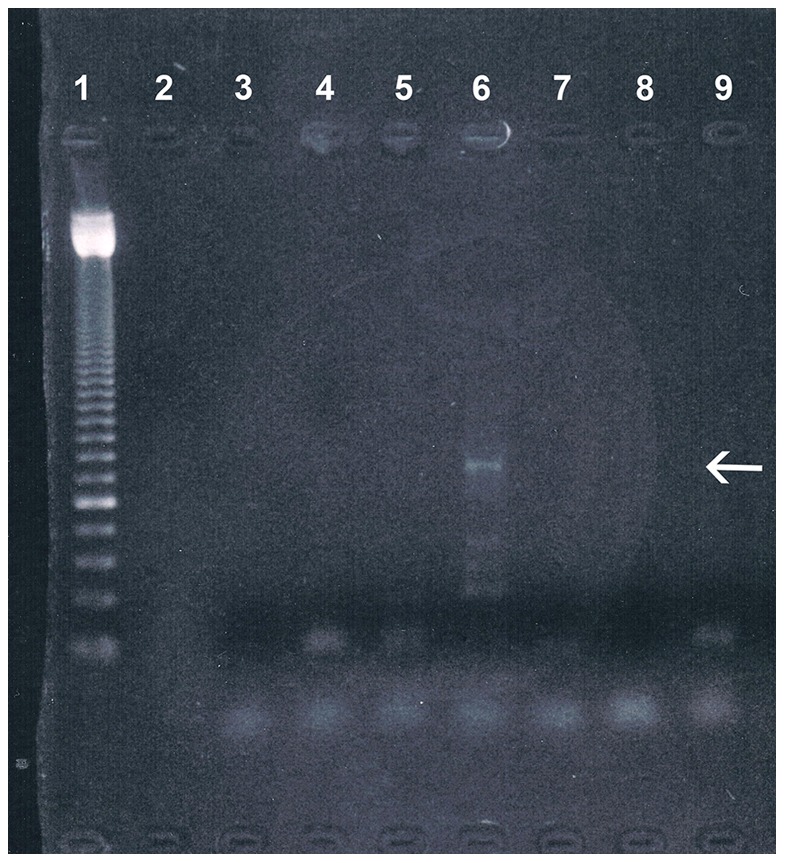
Molecular analysis of Trypanosoma cruzi ancient DNA. The agarose gel electrophoresis shows in lane 6 a positive amplicon of the expected size (arrow). (Lane 1: molecular weight standard; lanes 2–3: blank controls; lane 4–5: negative controls; lane 6–7: rectal wall tissue specimen of the mummy; lane 8–9: blank controls).

We succeeded in obtaining DNA as evidenced by a positive amplicon for the ubiquitous human beta-actin gene of the expected size of 202 bp (data not shown). The subsequent application of PCR primers specific for *Trypanosoma cruzi* revealed in one out of two extracts the expected amplicon size of 330 bp. Figure 8 illustrates that besides this amplicon three smaller fragments were generated most likely due to DNA degradation. All negative and blank extraction controls run in parallel remain negative. In an attempt for further molecular characterization, the amplicon was subjected to Sanger sequencing where we achieved a sequence of 74 bp length with high matching quality to *T. cruzi* sequences (Table I). The matching is described by an e-value of 2e-26. Thereby we provide an identity of 97% (query coverage 100%, gaps 0%) to the sequenced portion to the expected *T. cruzi* sequence (2 mismatches only). Using the BLASTn algorithm and nr databases the best score was obtained with the *Trypanosoma cruzi* spliced leader mini-exon repeat region (partial sequence), deposited under accession numbers AY367128.1, AY367127.1, AY367125.1, and U57984.1. The next best hit was obtained with accession number AY367124.1, providing an e-value of 2e-16, 89% sequence portion identity (66 out of 74 bases) and 0% gaps (data not shown).

### Stable Isotope Analysis

Most interestingly, the carbon isotope data in the hair show that the basic food plant of this individual was a C4-plant like maize as a characteristic crop for ancient Southern America [18]. Furthermore, the extraordinarily high nitrogen isotopic data of a maximum of 25.2‰ in the hair of that individual is strongly indicative for a marine diet. Similarly high values have been shown for Inca mariners in Northern Chile [19] and Southern Peru [20].

Over the ten month period analyzed, the delta^13^C values in the hair strand ranged from −12.5 to −11.0‰ with only a slight increase during the last two months period. In contrast, the delta^15^N data showed a significant variation over the tested time period and started to differ approximately two months before the death of the individual (Table II). The values declined by 1.7‰ at the delta^15^N.

### Molecular Analysis of Psychoactive Substances

The analyzed hair sample did not provide any measureable trace of cocaine or terahydrocannabinol (data not shown).

## Discussion

The interdisciplinary study of human mummified corpses of unknown provenance may provide surprising insight into the origin of the individual, but may also unravel unique features of the person’s life and/or death. The “outcome” of such studies strongly depends (i) on the material available (with mummies being much more informative than “only bone”), (ii) the condition of tissue preservation, but (iii) also on the available analytical techniques and accordingly the applied study strategy. Therefore, a stepwise combined application of various non-destructive, but eventually also destructive approaches is mandatory.

In the present study, we initially faced several problems: (i) where does the mummy come from? (ii) what can we say about her physical constitution? and (iii) is there any evidence for diseases and (iv) for her cause of death?

### The Origin of the Mummy

Obviously due to losses in the archives (probably during World War II) we initially only knew that our study object was in the hands of the Anatomical Institute of the Ludwig-Maximilians University, Munich since 1904; however, no records indicated its origin. Due to the brown colour, a descent from a moor in the surroundings of the Bavarian capital has previously been assumed [4]. However, the well preservation of soft and especially bone tissue (already seen on the few available radiographs in the 1970ies) as well the squat position and the style of the hair plait strongly argued against a local “bog” origin.

We proved instead a South American origin of the mummy based on several observations:

the stable isotope data of a mainly marine nitrogen and South American C4 plant (e.g. maize) diet is a very strong indicator for an origin of the individual from the coastal zone of Southern Peru (or Northern Chile). In particular, the mean carbon value of −12,0‰ fits very well to known hair data of South American mummies [18], [19]. The extraordinarily high nitrogen isotopic data of maximum 25,5‰ in the hair is well compatible with the values by Aufderheide et al. [19] who found nitrogen values determined from 23,7 to 27,3‰ in bone collagen of Inca mariners from Northern Chile dating to the same period as our individual. There are no European data sets available that might be compatible with our stable isotope results.
the morphological analysis of the tiny ropes fixing the hair of the long plaits are best compatible with camelid origin, additionally providing evidence for South American camelids (lama, alpaca).
The Wormian bone of the mummy (“os incae”) could be classified as (the rare) type III occipital Wormian bone [16] which is seen in South American populations in a frequency of approximately 8%, but which has not been found by Hanihara and Ishida [16] in a large series of Middle European (German) origin. Similarly, the skull deformation pattern seen on the CT scans of the occipital head are typical for South American descent [17].
The individual suffered from severe chronic Trypanosomiasis (Chagas disease) – a disease that is typical in South and Meso-America, while completely absent from present-day as well as ancient Europe.

In summary, we have clear evidence that the mummy stems from South America, most likely from the coastal or near-coastal zone of Southern Peru or Northern Chile. To this regard, it is of note that the Bavarian Princess Therese von Bayern (1850–1925), daughter of the Bavarian Prince regent Luitpold and well-known natural scientist at the ending 19^th^ century, undertook an “expedition” to Peru in 1898 from where she brought back two Peruvian mummies (according to her own records). However, the deposition of both mummies has not been recorded (or the records have been lost). It is, nevertheless, possible that one of those mummies was handed over the Anatomical Institute of Munich University [21].

### Anthropological and Paleopathological Data on the Living Conditions

The female died at young adult age, which could be estimated by incompletely fused vertebral apophyses to approximately 20 to 25 years. In consistency, her dental wear was low and she did not show major abrasion of joints and vertebra – as also seen histologically in the knee joint cartilage sample which revealed a delicate surface and normal chondrocytic lacunar distribution. There was only one small apical granuloma suggesting local caries with inflammation of the tooth root and resorption of the adjacent alveolar bone.

More interestingly, the mummy presented with significant thickening of several muscular tissues of thoracic and abdominal organs, in particular of the heart muscle and the small and large intestines including the rectum. The latter presented with distension of the lumen along with a strongly thickened tissue wall. These features are typically present in chronic infections by *Trypanosoma cruzi* (Chagas disease). In order to confirm this diagnosis our combined histological/immunohistological and molecular analysis proves an extensive infection by the parasite: both the intracellular inclusion of corpuscles of the expected size in the remnants of smooth muscle cells which react selectively and specifically with a monospecific antibody along with a specific molecular amplicon of the expected size and sequence homology support this diagnosis. Chagas disease is widespread in present day populations of South and Meso-America [22]. Several studies support the notion that this infection was also present in historic mummified tissue material [13]– [15] with the diagnosis suggested by typical visceromegaly and/or molecular identification of the pathogen. Therefore, we can add here a further case of that disease in South America. Most remarkably, the extent of rectal wall fibrosis suggests a chronic infection which is surprising in our 20 to 25 year old individual and which indicates an infection already in early infancy.

### Skull Trauma and Possible Evidence for its Origin

The most remarkable findings affected the skull. Despite an only minimal external skin lesion of the forehead the complete face and central skull bones are destroyed. The fact that the face still has retained an almost normal outer shape indicates that the bones were dislocated into the skull cavity post mortem. However, the slightly dehiscent skin lesion is indicative for a perimortem lesion since otherwise – at least after the mummification process was completed – any tear of the skin would have produced a larger tissue defect with significant dislocation of the defect margins. In consequence, the skin lesion must have occurred as long as the face bones were still in their anatomical position and the dislocation of the skull fragments must have occurred after decomposition of the brain and endocranial soft tissues. At that time, the natural mummification process must already have prevented the facial soft tissue from collapse into the “empty” mid-face hole.

Furthermore, the intactness of the mandible and the lack of any major bone injury (except some very obvious post-mortem fractures of isolated ribs on both sides, and the right clavicle and acromion) argue against a fall from significant height. Post mortem destruction of the skull by high pressure following burial, or other mechanical influence would have produced longitudinal or transversal fractures of the skull base, but not the type of lesion seen here. Therefore, a destruction of the skull long time after death is very unlikely.

With regard to the biomechanics of the severe mid-face injury, we assume that several massive beats with a blunt force must have hit the individual’s face from frontal position. The terrace-like margins suggest that the weapon was of a slightly round contour. Similar weapons have been identified in ancient South American armouries [23].

Perimortem lesions, very similar to those described here, have been reported recently in a South American male juvenile mummy (7 years old Aymara boy from the Titicaca region, Bolivia). However, the extent of the face fractures in this case is far less extensive than in our case [24]. Furthermore, there is ample evidence that South American religious belief covered ritual homicide – particularly of female infants (such as the very famous “Juanita”) or young females [25]. It remains highly speculative, although not impossible that our young lady was also subjected to such a ritual murder which must, however, have been executed with massive blunt force although there is an open debate as to trauma due to violence of occupational hazards [25]– [27]. Interestingly, the molecular search for psychoactive substances, such as cocaine, failed to show positive results.

### Reconstruction of the Final Months of Life

Finally, our stable isotope analysis provided us with important information upon the final months of life of the affected individual. Interestingly, we observed a significant and gradual decrease in the delta^15^N values by approximately 3,2‰ which starts to decrease about three months before the individuals death. This indicates a change in the person’s diet either due to a change in the available sea food due to seasonal reasons, or – more probably – by a move from the sea shore to the in-lands. Since in our case the delta^13^C values are relative steady over the last ten month we have good arguments to speculate that the carbon source must have been constant, although the delta^15^N differ over that time period. In consequence, we may think of a certain migration from the seashore to the inland within the last two months before death assuming a change in the protein source from seafood to a more terrestrial food source [28].

## Conclusion

Taken all data together, we provide circumstantial evidence that the mummy of the Bavarian Archaeological State Collection originates from South America, most probably from the coastal area of Southern Peru. Furthermore, the data indicate a change in food supply of the individual during her last two months suggesting a more terrestrial diet than sea food in this final period. The massive skull trauma indicates massive central blunt force that must have been acquired perimortally. The young age of approximately 20 to 25 years further supports the speculation that the individual suffered from ritual homicide – as previously seen repeatedly in South America. Finally, the individual suffered from Chagas disease – the result of chronic *Trypanosoma cruzi* infection – that obviously has been acquired in early infancy. Likewise, the life expectancy of the individual was low even without her final fatal course as a murder victim.

## Supporting Information

Text S1 **Histological and immunohistochemical investigation of mummy tissue specimens.**
(DOC)

Text S2 **Background of stable isotope analysis.**
(DOC)

Text S3 **Information of the aDNA analysis in the mummy biopsy taken from the rectal wall.**
(DOC)
