# Supplementary material for: Reconstructing the Life of an Unknown (ca. 500 Years-Old South American Inca) Mummy – Multidisciplinary Study of a Peruvian Inca Mummy Suggests Severe Chagas Disease and Ritual Homicide
Source: PLoS One. 2014 Feb 26;9(2):e89528. doi: 10.1371/journal.pone.0089528 (PMC3935882; doi:10.1371/journal.pone.0089528)
Supplement: Text S1 — Histological and immunohistochemical investigation of mummy tissue specimens. (DOC) [file pone.0089528.s001.doc]

**Supporting Information S1**

**Histological and immunohistochemical investigation of mummy tissue specimens**

The histological analysis of mummy tissue may provide substantial information on (i) the degree of tissue preservation, (ii) the identification of the tissue type (especially when the exact anatomical origin of a specimen is uncertain) and (iii) the presence of distinct pathological conditions. We therefore performed an extensive histological investigation on mummy tissue that was available by minimally invasive approach, i.e. that was rather easily accessible form the surface.

***1. Investigation of a bone/cartilage sample***

A full thickness sample of bone with overlying articular cartilage was removed from the right patella that was accessible from a defect caused by the loss of the thigh. The sample of c. 1 cm size was immediately after removal carefully mechanically cut into two halves. Both specimens were first rehydrated using a modified Ruffer solution [1] which was changed every day for 7 days until the samples were briefly post-fixed in 4% formaldehyde solution, pH 7.4 for another 24 hours.

Subsequently one half was subjected to a low temperature embedding into methyl methacrylate resin as previously established for bone biopsies [2] and which had been successfully tested on mummy bone tissue [3]. Therefore, the sample was transferred into 70% methanol at 4°C which was replaced by acetone (in the cold) after 4 hours. Following this dehydration process for another 4 hours, the sample was infiltrated with the acrylate again under cold temperature conditions for another 6 hours. Termination of the polymerization process was thereby achieved within 24 hours. The resulting bloc was used to prepare 2 µm thick undecalcified hard-tissue sections of bone and cartilage. These were subjected to Masson-Trichrome staining, toluidin blue staining and Giemsa-May Grünwald stains as routinely performed [2].

The second half of the original sample was transferred following the rehydration process into 0.1 M EDTA solution, pH 7.4, with gentle stirring and daily changes of the solution until complete decalcification which was achieved by about 14 days [4]. The tissue was then embedded into paraffin wax as routinely done by immersion into increasing alcohol concentrations, xylol and finally the wax at 60°C. The resulting paraffin wax bloc was used for the preparation of paraffin section which were stained for haematoxylin and eosin (H&E), Masson trichrome, Prussian blue, periodic acid-Schiff reagent (PAS) and alcian blue [4,5].

The sections were evaluated under the light microscope.

***2. Investigation of a rectal wall sample***

The full thickness specimen of the rectal wall that had been obtained by excision with a scalpel was again divided into two halves. One half was rehydrated as indicated before and subsequently embedded into paraffin wax. This procedure paralleled the above indicated technique. The resulting paraffin wax bloc was then used for the preparation of sections which were stained for haematoxylin and eosin (H&E), Masson trichrome and van Gieson connective tissue stains, Prussian blue and PAS staining [6,7].

The stained sections were evaluated under light microscopy.

***3. Immunohistochemical staining for Trypanosoma cruzi***

The paraffin wax bloc was additionally used for the immunostaining to detect T. cruzi. Appropriately deparaffinized tissue sections were shortly pretreated for antigen retrieval with 0.1% pronase, pH 6.0, and carefully washed. Subsequently, the primary antibody (anti-Trypanosoma cruzi LPG, No. AM01252SU-N, Acris GmbH, Herford, Germany) reacted with the pretreated sections for 20 mins., and the imunoreaction was then developed – following washing steps – with the alkaline phosphatase- anti-alkaline phosphatase secondary antibody staining system (DAKO, Hamburg, Germany). The reaction product was visualized with fast red and evaluated under the light microscope [4, 7].

For control purposes, bovine serum was applied instead of the primary antibody to otherwise identically treated tissue sections and served as negative controls.

**References cited**

1. Ruffer MA (1910) Remarks on the histology and pathological anatomy of Egyptian mummies. Cairo Scientific J. 4, 40.

# 2. Lebeau A, Muthmann H, Sendelhofert A, Diebold J, Löhrs U (1995) Histochemistry and immunohistochemistry on bone marrow biopsies. A rapid procedure for methyl methacrylate embedding. Path. Res. Pract. 191: 121-129.

3. Nerlich A, Parsche F, Wiest I (1995) Untersuchungen des Kollagens an historischem Knochen- und Mumiengewebe. Osteologie 4: 44-52.

4. Nerlich AG, Parsche F, Kirsch T, Wiest I, von der Mark K (1993) Immunohistochemical detection of interstitial collagens in bone and cartilage tissue remnants in an infant Peruvian mummy. Am J Phys Anthropol. 91: 279-85.

5. Nerlich, A.G., Zink, A. (2003). Histopathology of knee menisci in ancient Egyptian mummies. Journal of Paleopathology 15: 5-12

6. Nerlich AG, Parsche F, Wiest I, Schramel P, Löhrs U (1995) Extensive pulmonary haemorrhage in an Egyptian mummy. Virchows Arch. 427: 423-9

7. Nerlich A, Parsche F, Wiest I (1999) Immunhistochemische Untersuchungen an einer Trockenmumie aus der Pfarrkirche von Altlichtenwarth in Niederösterreich. Fundber. Österreich 38: 697-700
