# Supplementary material for: Reconstructing the Life of an Unknown (ca. 500 Years-Old South American Inca) Mummy – Multidisciplinary Study of a Peruvian Inca Mummy Suggests Severe Chagas Disease and Ritual Homicide
Source: PLoS One. 2014 Feb 26;9(2):e89528. doi: 10.1371/journal.pone.0089528 (PMC3935882; doi:10.1371/journal.pone.0089528)
Supplement: Text S2 — Background of stable isotope analysis. (DOC) [file pone.0089528.s002.doc]

**Supporting Information S2**

**Background of stable isotope analysis**

***1. Background of the stable isotope analysis of nitrogen isotopes***

The ratio of δ^15^N is related to the trophic position of any specific organism within the local food web. Nitrogen can originate from plant proteins with usually low levels of the ^15^N isotope which, however, can accumulate by re-integration into an organism at a higher level of trophy. Animal nitrogen sources accordingly show higher levels of trophy than plant sources. Likewise, while many plants show δ^15^N values of approximately 3 %o, herbivorous animals reveal values of approx. 9%o [1].

The level of the trophic position is usually higher in aquatic food due to the presence of zooplankton and insect pray at the base of this food chain [2]. As a result δ^15^N ratios of fish are often significantly higher than those of terrestrial animals [3], despite the fact that fish may consume a very diverse mixture of aquatic food sources containing variable δ^15^N ratios. In addition to these general trends, δ^15^N values of plants and animals may differ between various regions [3]. This may be the result of influences by climate [4], physiology [4], [5] and subsistence techniques such as crop manufacturing or salt-marsh grazing [6], [7]. These variables generate inter-regional differences in human δ^15^N values that are not related to diet per se, which complicates isotopic comparisons of diet across populations.

In case of exceedingly high ^15^N isotope levels – such as in the individual under investigation in this report – the high δ^15^N value may be very indicative for the origin of the respective individual: Extremely high levels of δ^15^N are seen in populations with a dominance of sea food nutrition (South African and South American coast line populations) since here the extraordinarily high level of nitrogen isotope trophy results for the nitrogen isotope rich food supply of sea fish, seals and walruses which may serve as a main nutritional source.

Therefore, in the present case, a (at least near) coastal line origin of the individual is highly presumable.

***2. Background of the carbon isotope analysis***

The ratio obtained using carbon stable isotopes, mainly ^12^C / ^13^C, is assessed to determine the relative consumption of two classes of plants: so-called “C3 plants” include most vegetables, wheat, and barley, while the “C4 plants” are made up by millet, maize and other tropical grasses [8]. The main differences between these two classes of plants are different photosynthetic pathways through which carbon is fixed from the atmosphere into the plant tissue [9]. The major source of carbon for terrestrial ecosystems is atmospheric CO_2_ with a δ^13^C ratio of approximately – 8%o [10]. The photosynthetic pathway of C3 plants (called Calvin cycle) preferentially incorporates the lighter isotope ^12^C during metabolism and discriminates against ^13^C. Consequently, C3 plant tissues are considerably depleted compared to CO_2_ exhibiting values of -20,0 to -35,0 %o [11]. C4 plants use a different pathway (called the Hatche-Slack pathway) to metabolize carbon. This does not discriminate as stringently against the heavier isotope leaving tissues ^13^C-enriched compared to C3 plants. The δ^13^C values of C4 plants range from – 9,0%o to -14,0 %o and do not overlap those of C3 plants [11].

Marine plants metabolize carbon present in oceans via the C3 photosynthetic pathway. The major source of carbon in marine ecosystems is dissolved inorganic carbon (including carbonic acid and CO2) [12]. Particulate organic matter (including algae) is another source of carbon in marine environments with δ^13^C values generally ranging from -18.5 to -22.0%o [12]. The δ^13^C ratios of plants and animals occupying marine niches fall between these values and marine fish δ^13^C values can range from approximately -11.0 to -19.0 %o [13].

The δ^13^C composition of plants in freshwater ecosystems is highly variable. Unlike in terrestrial or marine environments where carbon derives primarily from a single source (atmospheric CO2 on land and dissolved carbonate in oceans), carbon composition in freshwater environments is heavily influenced by isotopic equilibration between water and atmosphere and also by decomposing organic matter. Freshwater fish consequently exhibit a broad range of δ^13^C values, but studies suggest their values are usually depleted due to the low δ^13^C ratios of aquatic plants [14], [15]. Depletion in δ^13^C ratios of human bone below what might be expected of a terrestrial diet (more negative than approximately -22.0%o the low-end of what could be explained by a C3-only terrestrial diet) suggests fresh water fish consumption.

In the present case the high value for the carbon isotope ^13^C favours a C4-derived plant diet of the individual. As described above, this pattern is restricted to maize and some other tropical grasses. Thereby, the most probable nutritional source comes from the New World.

Taking the isotope values form nitrogen and carbon isotopes together, the typical pattern localizes the individual to a coastal area of Southern America, i.e. Southern Peru / Northern Chile. In those regions, previous studies have identified a similar istopic pattern as in our present study.

**References cited:**

1.[Schoeninger MJ](http://www.ncbi.nlm.nih.gov/pubmed?term=Schoeninger MJ%5BAuthor%5D&cauthor=true&cauthor_uid=1443094), DeNiro MJ (1984) Nitrogen and carbon isotopic composition of bone collagen from marine and terrestrial animals. Geochim. Cosmochim. Acta 48: 625-639.

2. France RL (1994) Nitrogen isotopic composition of marine and freshwater invertebrates. Mar. Ecol. Prog. Ser. 115: 205-207

3. DeNiro MJ, Epstein S (1981) Influence of diet on the distribution of nitrogen isotopes in animals. Geochim. Cosmochim. Acta 45: 341-351.

4. Ambrose SH, Sikes NE (1991) [Soil carbon isotope evidence for holocene habitat change in the kenya rift valley.](http://www.ncbi.nlm.nih.gov/pubmed/17793482) Science 253: 1402-5.

5. Fuller BT, Molleson TI, Harris DA, Gilmour LT, Hedges RE (2006) [Isotopic evidence for breastfeeding and possible adult dietary differences from Late/Sub-Roman Britain.](http://www.ncbi.nlm.nih.gov/pubmed/16229026)

Am J Phys Anthropol. 129: 45-54.

6. Bogaard A, Heaton THE, Poulton P, Merbach I (2007) The impact of manuring on nitrogen isotope ratios in cereals: archaeological implications for reconstruction of diet and crop management practices. J. Archaeol. Sci. 34: 335-343.

7. Britton K, Müldner G, Bell M (2008) Stable isotope evidence for salt-marsh grazing in the Bronze Age Severn Estuary: implications for paleodietary analysis at coastal sites. J. Archaeol. Sci. 35: 2111-2118

8. Smith BN, Epstein S (1971) [Two categories of c/c ratios for higher plants.](http://www.ncbi.nlm.nih.gov/pubmed/16657626) Plant Physiol. 47: 380-4.

9. Bender MM (1968) Mass spectrometric studies of carbon 13 variations in corn and other grasses. Radiocarbon 10: 468-472.

10. Marino BD, McElroy MB (1991) Isotopic composition of atmospheric CO_2_ inferred from carbon in C4 plant cellulose, Nature 349: 127-131.

11. Katzenberg AM (2000) Stable isotope analysis: a tool for studying past diet, demography, and life history. In: Katzenberg AM, Saunders SR (Ed) Biological Anthropology of the Human Skeleton. Wiley-Liss Inc. New York

12. Hoefs J (2004) Stable isotope geochemistry. Springer-Verlag, Berlin.

13. Barrett J, Johnstone C, Harland J, Van Neer W, Ervynck A, Makowiecki D, Heinrich D, Hufthammer AK, Enghoff IB, Amundsen C, Christiansen JS, Jones AKG, Locker A, Hamilton-Dyer S, Jonsson L, Lougas L, Roberts C, Richards MP (2008) Detecting the medieval cod trade: a new method and first results. J. Archaeol. Sci. 35: 850-861.

14. Dufour E, Herve B, Mariotti A (1999) Paleodietary implications of isotopic variability in Eurasian lacustrine fish. J. Archaeol. Sci. 26: 617-617.

15. Pazdur A, Goslar T, Pawlyta M, Hercman H, Gradzinski M (1999) Variantions of isotopic composition of carbon in the Karst environment from southern Poland, present and past. Radiocarbon 41: 81-97
