# Supplementary material for: Reconstructing the Life of an Unknown (ca. 500 Years-Old South American Inca) Mummy – Multidisciplinary Study of a Peruvian Inca Mummy Suggests Severe Chagas Disease and Ritual Homicide
Source: PLoS One. 2014 Feb 26;9(2):e89528. doi: 10.1371/journal.pone.0089528 (PMC3935882; doi:10.1371/journal.pone.0089528)
Supplement: Text S3 — Information of the aDNA analysis in the mummy biopsy taken from the rectal wall. (DOC) [file pone.0089528.s003.doc]

**Supporting Information S3**

**Information on the aDNA analysis in the mummy biopsy taken from the rectal wall**

***1. Tissue preparation and DNA extraction***

The tissue sample was examined for the presence of *Trypanosoma cruzi* nucleic acids according to previously established protocols specifically designed for aDNA research [e.g. 1], [2]. The analysis was performed independently in two different laboratories (München and Tübingen) which are each equipped with separate rooms dedicated to aDNA research and multiple equipment for pre- and post-PCR procedures.

The sample was mechanically disintegrated and finally crushed using a sterile pestle and mortar, weighed and subjected to proteinase K treatment (0.25 mg/ml) with incubation at room temperature and gentle shaking on a rotary mixer for two days [3]. In order to increase the amount of isolated ancient DNA we further applied three cycles of freeze-thawing as previously described [2].

Following centrifugation for 15 min at 3000 g, 0.5 ml of the supernatant was removed and 1 ml guanidine isothiocyanate solution and diatomaceous earth [4] were added. After incubation on a rotatory mixer for another 2 hours, the diatomaceous earth was pelleted by centrifugation and washed twice with 70 % ethanol and once with acetone. The DNA was eluted with 80 µl sterile water. Finally, another washing and concentration step was performed with Microcon-30 filters (Millipore, Bedford, MA). The resulting DNA (c. 1µl fluid) was then diluted with sterile water to a volume of 10 µl. This was done in order to provide sufficient template volume for repeated PCRs (including subsequent Sanger sequence analysis). The total DNA concentration ranged between 20 – 40 ng/µl.

***2. Precautions to avoid contamination and methods to ensure authentication of DNA amplification***

Several precautions were taken to avoid contamination during the extraction procedure and in the PCR reactions. The extraction, PCR and post-PCR analyses were all conducted in separated rooms of the München buildings, where no studies of modern trypanosomal DNA have been performed before. All reagents were purchased as DNAse and RNAse-free Molecular Biology Grade chemicals or autoclaved when appropriate. No positive PCR controls were used. Disposable gloves were worn during all different procedures and changed frequently. Sterile aerosol protection tips (Safeseal tips, Biozym, Hess. Oldendorf, Germany) were exclusively used to avoid cross-contamination. At least, two extraction blanks were always performed in the same procedure and additionally a PCR blank control was included in each PCR reaction, containing no DNA template. Finally, an aliquot of the specimen was analysed in two different institutions (Munich – Tübingen). Both institutes employed similar analytical procedures in parallel and obtained congruent results.

***3. Amplification of human aDNA as control reaction***

To test whether amplifiable DNA was present in the samples and to ascertain that the PCR was not inhibited, a 202 bp segment of the human β-actin gene was amplified in parallel as previously done in other studies [1], [2], [8]. The PCR mixture was prepared as described above. The following amplification protocol was used: 10 min at 95 °C, 45 cycles of 94 °C for 1 min, 60 °C for 3 min and 72 °C for 3 min, and final extension at 72 °C for 8 min.

***4. Amplification of kinetoplastic aDNA of Trypanosoma cruzi***

For the specific amplification of trypanosomal DNA [5] we used a primer pair targeting a 330 base pair (bp) segment of the kinetoplastic mino-exon of *T. cruzi* covering both *T. cruzi* I and II genotypes [6] since previous studies had shown that shorter sequences proved not to show enough specificity of the reaction in order to safely identify *T. cruzi* [7].

The primers used were: (5´- CCCCCCTCCCAGGCCACACTG) and (5´- CCTGCAGGCACACGTGTGTGTG): The PCR reaction mix contained 10 mM Tris-HCl (pH 8.3), 50 mM KCl, 1.5 mM MgCl_2_, 200 µM of each deoxynucleotide trisphosphate (Amersham Pharmacia, Uppsala, Sweden), 1 µM of each primer, 0.025 U/µl AmpliTaq Gold (PE Biosystems, Foster City, CA) and 0.5 µL extracted DNA to a final volume of 20 µl. PCR-conditions were as follows: 10 min at 95 °C followed by 45 cycles of 94 °C for 1 min, 66 °C for 1 min and 72 °C for 1 min. After the final cycle another 8 min at 72 °C were added.

***5. Identification of PCR products***

The PCR products were electrophoresed on a 4 % agarose gel and visualised on a UV-screen after staining with ethidium bromide.

***6. Sequence analysis of PCR products***

The nucleotide sequences of the PCR products were determined by direct sequencing: After electrophoresis on a 4% low-melting-point agarose gel, the respective fragment of the PCR reaction was eluted with a purification kit (Freeze’n Squeeze, Bio-Rad, Hercules, CA). With the eluted DNA, cycle sequencing was performed with dye terminator cycle sequencing chenistry (PE Biosystems). Automated sequencing was performed on an ABI PRISM 310 Genetic Analyzer (PE Biosystems).

**References cited**

# 1. Nerlich AG, ****Schraut B, Dittrich S, Jelinek T, Zink AR (2008)**** *Plasmodium falciparum* in Ancient Egypt. Emerg Infect. Dis. 14: 1317-1318.

2. Zink AR, Sola C, Reischl U, Grabner W, Rastogi N, Wolf H, Nerlich AG (2003) Characterization of Mycobacterium tuberculosis complex DNAs from Egyptian mummies by Spoligotyping. J. Clin. Microbiol. 41: 359-167.

3. Boom R, Sol CJA, Salimans MMM, Jansen CL, Wertheim-van Dillen PME, van der Noordaa J. Rapid and simple method for purification of nucleic acids. J Clin Microbiol 1990; 28: 495-503.

4. Evison MP, Smillie DM, Chamberlain AT. Extraction of single-copy nuclear DNA from forensic specimens with a variety of postmortem histories. J Forensic Sci 1997; 42: 1032-1038.

5. Luchtan M, Warade C, Weatherly DB, Degrave WM, Tarleton RL, Kissinger JC (2004) TcruziDB: an integrated Trypanosoma cruzi genome resource. Nucl. Acid Res. 32: D344-D346.

6. Fernandes A, Iniguez AM, Lima VS, Mendonca de Souza SMF, Ferreira LF, Vicente ACP, Jansen AM (2008) Pre-Columbian Chagas disease in Brazil: Trypanosoma cruzi I in the archaeological remains of a human in Peruacu Valley, Minas Gerais, Brazil. Mem. Inst. Oswaldo Cruz 103: 514-1516.

7. Souto RP, Fernandes O, Macedo AM, Campbell DA, Zingales B, Souto RP (1996) DNA marker define two major phylogenetic lineages of Trypanosoma cruzi. Mol. Biochem. Parasitol. 83: 141-152.

8. Ghossein RA, Ross DG, Salomon RN, Rabson AR. A search for mycobacterial DNA in sarcoidosis using the polymerase chain reaction. Am J Clin Pathol 1994; 101: 733-737.
